# *Escherichia hermannii* Infections in Humans: A Systematic Review

**DOI:** 10.3390/tropicalmed4010017

**Published:** 2019-01-21

**Authors:** Petros Ioannou

**Affiliations:** Department of Internal Medicine & Infectious Diseases, University Hospital of Heraklion, Heraklion, Crete PC 71500, Greece; p.ioannou@med.uoc.gr; Tel.: +30-2810-392728, Fax: +30-2810-392359

**Keywords:** *Escherichia hermannii*, bacteremia, UTI, urinary tract infection

## Abstract

*Eshcerichia hermannii* is a member of the Enterobacteriaceae, first described in 1982 and reclassified as a distinct species in the *Escherichia* genus after identifying biochemical and genomic differences from *E. coli.* It is a rare cause of human infections and is supposed to be a co-infector rather than an autonomous cause of infection. The aim of this systematic review was to record and evaluate all available evidence regarding human infections by *E. hermannii*. A systematic review of PubMed (through 21 December 2018) for studies providing epidemiological, clinical, and microbiological information, as well as treatment data and outcomes of *E. hermannii* infections was performed. A total of 16 studies, containing data of 17 patients, were eventually included in the analysis. The most common *E. hermannii* infections were bacteremias, urinary tract, and central nervous system infections. The complication rate, like the occurrence of sepsis, was high. Cephalosporins and aminoglycosides were the most common agents used for treatment. This systematic review describes bacterial infections by *E. hermannii* and provides information on the epidemiology, clinical presentation, antibiotic resistance, treatment, and outcomes associated with these infections.

## 1. Introduction

*Escherichia hermannii* is a gram-negative bacterium that belongs in the family of Enterobacteriaceae and was first described in 1982 [1]. It had been formerly known as enteric group 11 of *E. coli* but was later on reclassified as a distinct species in the *Escherichia* genus after identifying unique genomic features that allowed discrimination from *E. coli* [2]. In the laboratory, *E. hermannii* can be distinguished from *E. coli* because of the production of a yellow pigment. *E. hermannii* has been a rare cause of human infections and is supposed to be mostly a co-infector in polymicrobial infections and not considered truly pathogenic [3]. However, there is evidence of the pathogenicity of this bacterium, which seems to be able to cause infections even in immunocompetent, non-predisposed individuals [4].

The purpose of this study was to systemically review all published cases of *E. hermannii* infections and describe the epidemiology, microbiology, antimicrobial susceptibility, treatment, and outcomes of these human infections.

## 2. Methods

### 2.1. Data Search

For this review, the Preferred Reporting Items for Systematic Reviews and Meta-analyses (PRISMA) guidelines were adopted [5]. Eligible studies were identified through a search of PubMed MEDLINE with the following text-words: Escher*[tw] AND herman*[tw]. The day of the last search was 21 December 2018.

### 2.2. Study Selection

Studies were included in the analysis if they met the following criteria: (1) Published in English and (2) reported data on patients’ clinical characteristics, microbiology, antimicrobial susceptibility, treatment, and outcomes. Studies with the following criteria were excluded from the analysis: (1) Secondary research papers (e.g., reviews), editorials, and papers not reporting results on the primary research; (2) studies not in humans; and (3) studies on colonization but not infection by *E. hermannii*. The titles of the resulting references along with the studies’ abstracts were screened using Rayyan [6]. Then, the full text articles were retrieved and rescreened for potentially relevant articles. Reference lists of the included studies were searched for relevant articles.

### 2.3. Endpoints

This study’s endpoint was to record the type of *E. hermannii* infections included in the literature as well as the patient characteristics for different type of infections, the microbiological and antimicrobial susceptibility data on *E. hermannii* infections, and their treatment and outcomes.

### 2.4. Data Extraction and Definitions

Data from each eligible study were extracted based on study type, year of publication, and country; patient demographic data (age and gender); patient’s relevant medical history (diagnosis of cancer, autoimmune disease, and immunosuppression); infection data, microbiology, and antimicrobial susceptibility (predisposing conditions, such as the presence of a central venous catheter (CVC), infection site, and presence of complications); treatment administered for the infection; and outcomes (i.e., cure or death). The relation of death to the index infection was reported according to the study authors. The complications recorded included any organ dysfunction or clinical deterioration that was considered by the authors to be related to the *E. hermannii* infection.

## 3. Results

### 3.1. Literature Search

**Figure 1:**
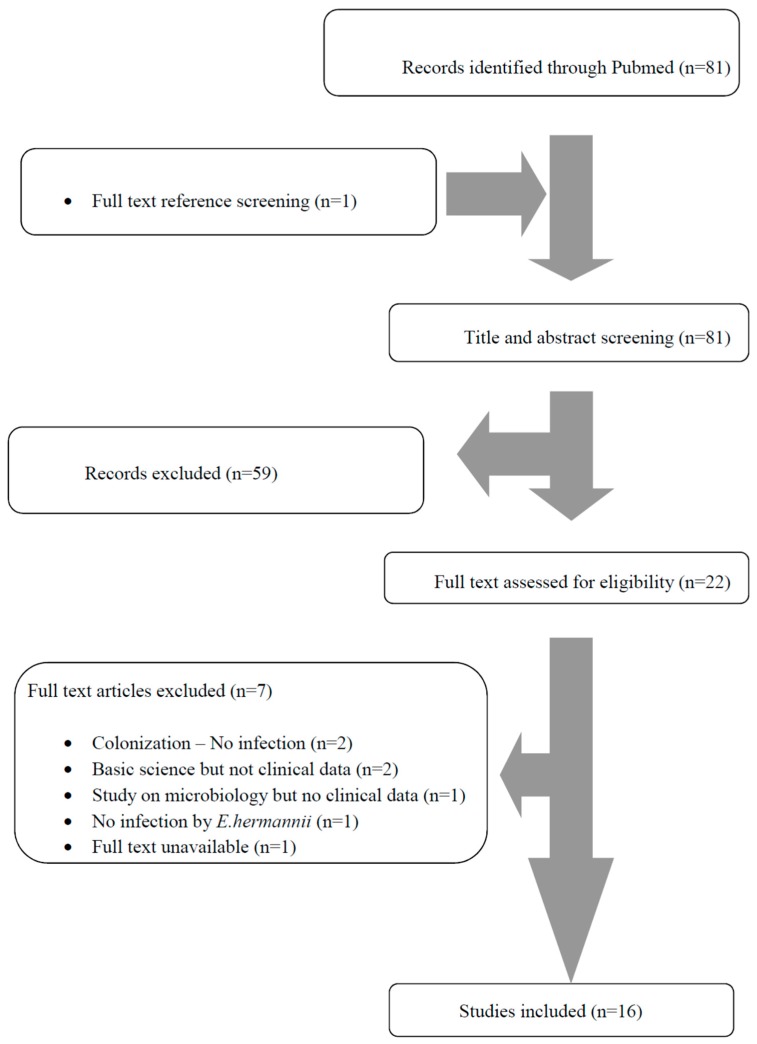
The Preferred Reporting Items for Systematic Reviews and Meta-analyses (PRISMA) flow diagram.

A total of 81 articles from PubMed were screened. After reviewing the titles and abstracts, 22 articles were selected for full-text review. From them, seven were excluded: two had data only on colonization by *E. hermannii*, four were not clinical (two were on basic research, and two had microbiological data only), two did not involve humans, and there was one article whose full text was unavailable. One additional study was found by screening the articles’ references. Finally, 16 met the study criteria [3,4,7,8,9,10,11,12,13,14,15,16,17,18,19,20]. Additional information was kindly provided by the corresponding author of one study [16]. The review process is graphically presented in Figure 1.

### 3.2. Included Studies’ Characteristics

The 16 studies that were finally included in this analysis involved a total of 17 patients with 7 studies conducted in Europe, 5 in North America, and 4 in Asia. The final sum included 12 case reports, 2 case series, and 2 microbiology studies.

### 3.3. Epidemiology, Microbiology, Antimicrobial Resistance Patterns, Treatment, and Outcomes of E. hermannii Infections

The patients’ ages rang ed from neonates to 80 years, with a median age of 41.5 years; 76.9% (when data were available) were male. The most common infections were bacteremias in 52.3% (9 out of 17 patients), urinary tract infection (UTI) in 23.5% (4 patients), central nervous system infection in 17.6% (3 patients), and gastrointestinal infections in 11.8% (2 patients), and the rest were abdominal cavity infection (peritonitis), conjunctivitis, and skin and soft tissue infection (SSTI) in 5.9% (1 patient each). Among the reported patients’ history, 33.3% had a central venous catheter (4 out of 12 with available data), 21.4% had chronic kidney disease (CKD) on hemodialysis (3 out of 14 with available data), 7.1% were immunosuppressed due to organ transplantation (1 out of 14 with available data), 7.1% had an active malignancy on chemotherapy (1 out of 14 with available data), and 7.1% had AIDS (1 out of 14 with available data).

An antibiogram was available in 82.4% (14 out of 17 patients with available data). All *E. hermannii* strains were penicillin resistant (13 out of 13 with available data), while 28.6% (2 out of 7 with available data) were resistant to the combination of piperacillin with tazobactam, 28.6% (2 out of 7 with available data) were resistant to tetracycline, 15.4% were cephalosporin resistant (2 out of 13 with available data), 12.5% (1 out of 8 with available data) were resistant to the combination of trimethoprim with sulfomethoxazole, 10% (1 out of 10 with available data) were resistant to carbapenems, 9.1% (1 out of 11 with available data) were quinolone resistant, and 7.7% (1 out of 13 with available data) were resistant to aminoglycosides. The extended spectrum beta lactamase phenotype (ESBL) was detected in 9.1% of cases (1 out of 11 with available data). A concomitant infection was present in 23.5% (4 out of 17 cases with available data). Of all patients, 60% (9 out of 15 with available data) were feverish, 53.3% (8 out of 15 with available data) were septic, 26.7% (4 out of 15 with available data) had organ dysfunction 13.3% (2 out of 15 with available data) developed shock, and 21.4% (3 out of 14 with available data) needed intensive care unit (ICU) care.

Treatment for *E. hermannii* infections consisted of cephalosporins in 41.7% (5 out of 12 with available data), aminoglycosides in 41.7% (5 out of 12 with available data), quinolones in 33.3% (4 out of 12 with available data), the combination of piperacillin and tazobactam in 25% (3 out of 12 with available data), carbapenems in 16.7% (2 out of 12 with available data), the combination of trimethoprim with sulfomethoxazole in 16.7% (2 out of 12 with available data), and the combination of amoxicillin with clavulanate in 6.3% (1 out of 16 with available data). Surgical drainage was performed in 14.3% (2 out of 14 cases with available data), and a central venous catheter was removed in all cases where a bacteremia had developed in a patient with a central venous catheter (4 out of 4 cases). The duration of the treatment varied from a minimum of 3 to a maximum of 56 days, and the median was 14 days. A clinical cure was achieved in 83.3% of cases (10 out of 12 patients with available data), and the overall mortality was 16.7% (2 out of 12 patients with available data), but the mortality attributed directly to the *E. hermannii* infection was 8.3% (1 patient).

### 3.4. Bacteremias

Among the 16 *E. hermannii* infections studies, 9 (56.3%) reported bacteremias, accounting for 9 patients out of 17 (52.3%) in total [8,9,12,13,14,17,18,19,20]. Among the patients with bacteremia, 62.8% were male (7 out of 9 cases) with median age of 40 years. Among the reported patients’ history, 57.1% had a central venous catheter (4 out of 7 with available data), 33.3% had CKD on hemodialysis (3 out of 9 with available data), 11.1% were immunosuppressed due to organ transplantation (1 out of 9 with available data), and 11.1% had an active malignancy on chemotherapy (1 out of 9 with available data).

An antibiogram was present in 88.9% (8 out of 9 patients with available data). All *E. hermannii* strains were penicillin resistant (7 out of 7 with available data), while 25% (1 out of 4 with available data) were resistant to the combination of piperacillin with tazobactam, 25% (1 out of 4 with available data) were resistant to tetracycline, 25% (1 out of 4 with available data) were resistant to carbapenems, 16.7% (1 out of 6 with available data) were quinolone resistant, and 14.3% were cephalosporin resistant (1 out of 7 with available data). A concomitant infection was present in 22.2% (2 out of 9 cases with available data). Of all patients, 77.8% (7 out of 9 with available data) were feverish, 66.7% (6 out of 9 with available data) were septic, 22.2% (2 out of 9 with available data) had organ dysfunction, 11.1% (1 out of 9 with available data) developed shock, and 22.2% (2 out of 9 with available data) needed ICU care.

For the treatment of *E. hermannii* infections, aminoglycosides were used in 50% (4 out of 8 with available data), piperacillin and tazobactam in 37.5% (3 out of 8 with available data), cephalosporins in 25% (2 out of 8 with available data), quinolones in 25% (2 out of 8 with available data), the combination of trimethoprim with sulfomethoxazole in 25% (2 out of 8 with available data), carbapenems in 12.5% (1 out of 8 with available data), and the combination of amoxicillin with clavulanate in 12.5% (1 out of 8 with available data). The duration of treatment varied from a minimum of 3 to a maximum of 14 days, and the median was 14 days. A clinical cure was achieved in 85.7% of cases (6 out of 7 patients with available data), and the overall mortality was 14.3% (1 out of 7 patients with available data) with the mortality attributed directly to the *E. hermannii* infection. Table 1 shows the characteristics of patients with *E. hermannii* bacteremias.

### 3.5. Urinary Tract Infections

Among the 16 *E. hermannii* infections studies, 4 (25%) reported UTIs, accounting for 4 patients out of 17 (23.5%) in total [4,10,15,18]. Among the patients with a UTI, 66.6% were male (2 out of 3 cases with available data) with a median age of 54 years. Among the reported patients’ history, 25% were immunosuppressed due to organ transplantation (1 out of 4). A concurrent infection of the central nervous system was noted in 25% (1 out of 4 patients), and bacteremia was noted in 25% (1 out of 4).

All patients had an antibiogram available. All *E. hermannii* strains were penicillin resistant (4 out of 4), while 50% (1 out of 2 with available data) were resistant to the combination of piperacillin with tazobactam, 33.3% (1 out of 3 with available data) were resistant to cephalosporins, 33.3% (1 out of 3 with available data) were quinolone resistant, and 33.3% were aminoglycoside resistant (1 out of 3 with available data). Importantly, the ESBL phenotype was detected in 33.3% (1 out of 3 patients with available data). A concomitant infection was present in 25% (1 out of 4 cases). Of all patients, 66.7% (2 out of 3 with available data) were feverish, 66.7% (2 out of 3 with available data) were septic, 33.3% (1 out of 3 with available data) had organ dysfunction, and none (0 out of 3 with available data) developed shock or needed ICU care.

Treatment for *E. hermannii* infections consisted of a cephalosporin in 66.7% (2 out of 3 cases), the combination of trimethoprim and sulfomethoxazole in 66.7% (2 out of 3 cases), aminoglycosides in 33.3% (1 out of 3 with available data), quinolones in 33.3% (1 out of 3 with available data), and the combination of amoxicillin with clavulanate in 25% (1 out of 4). A clinical cure was achieved in 100% of cases (3 out of 3 patients with available data). Table 2 shows the characteristics of patients with *E. hermannii* urinary tract infections.

## 4. Discussion

During the last decades, several members of the Enterobacteriaceae have been reclassified as unique species among the *Escherichia* genus, such as *E.vulneris, E.blattae, E.fegusonii,* and *E. hermannii*, based on intra-species DNA homology, guanine cytosine content, genome size, and biochemical tests [1,7]. *E. hermannii* can be differentiated by *E. coli* due to different biochemical characteristics, like a positive reaction to potassium cyanate, the fermentation of cellobiose, and the production of a yellow pigment [1]. In the last decades, more than 17 cases of human infections by *E. hermannii* have been reported, and this bacterium is generally considered to be one of low pathogenicity that accompanies other microorganisms during an infection [2,9,19]. This systemic review provides thorough information on epidemiology, clinical characteristics, treatment, and outcomes of *E. hermannii* infections. Bacteremia and urinary tract infection were the most common infections by *E. hermannii*.

Bacteremia, as well as the rest of the infections by *E. hermannii* in this analysis, has a male predilection, even though the small number of patients implies that caution is needed in drawing definite conclusions. The presence of a central venous catheter, most commonly in the context of CKD on hemodialysis, was present in more than half of the patients with *E. hermannii* bacteremia. Since there is evidence in the literature suggesting *E. coli* as the most common source of gram-negative bacteremia in patients on hemodialysis [21,22], the association of *E. hermannii* with hemodialyzed patients is reasonable. Antimicrobial resistance was not worrisome, since even though all *E. hermannii* strains are resistant to penicillin, and there were some strains resistant to all beta-lactams or to quinolones, no multidrug resistant strains were noted. The infection severity was notable, with the majority of the patients developing sepsis and an important proportion requiring ICU care, which reminds us of the severity of *E. coli* bacteremia [23].

Urinary tract infections by *E. hermannii* were the second most common infections after bacteremias, also carrying important morbidity, since most patients developed sepsis, yet all patients survived. Interestingly, in the case of one patient, the ESBL phenotype was detected in the antibiogram, implying that even though *E. hermannii*’s antibiotic resistance seems not to be burdensome, vigilance is required since there could be a potential for the development of multidrug resistance.

In this systematic review, *E. hermannii* was found to be a co-infector in less than 25% of infections, implying that the classic belief that *E. hermannii* is a colonizer microorganism with low pathogenicity is not correct [2,9,19]. Interestingly, when *E. hermannii* causes an infection on its own, it seems to be virulent enough to cause sepsis, just like *E. coli*. Thus, it could be that this microorganism is not a non-pathogenic one but a rare one.

The present systematic review has certain limitations that should be acknowledged. First of all, it mostly consists of case reports and case series. Thus, results should be read cautiously, as case reports are descriptions of unusual presentations while the usual ones may be underrepresented in a systematic review consisting of such studies. For example, it could be reasonable for someone to expect diarrhea to be the most common presentation of an *E. hermannii* infection, yet such a finding would not be easily publishable—even though there are a few reports [1,7]—and this site of infection would definitely be underrepresented. However, the present methodology was the only reliable way to systematically study *E. hermannii* infections [24]. If case reports and case series were excluded, as other investigators have done in other cases [25], there would be no studies left for inclusion. On the other hand, since *E. hermannii* infections are rare, one would expect this study to be representative enough, maybe with the exception of gastrointestinal infections. Thus, all informative cases reliably demonstrating the nature of *E. hermannii* infections have been included in this analysis.

In conclusion, this systematic review presents the epidemiology, clinical characteristics, microbiology, antimicrobial susceptibility, treatment, and outcomes of infections by *E. hermannii* with important clinical implications. Physicians should become familiar with these infections, since they carry considerable morbidity.

## Figures and Tables

**Table 1:** Characteristics of patients with *E. hermannii* bacteremias. The values show the cases among patients with available data.

Characteristic	Value
Male, *n* (%)	7 out of 9 (77.8%)
Age, median (IQR) in years	40 (16–64)
**Medical history**	
Central venous catheter, *n* (%)	4 out of 7 (57.1%)
Chronic kidney disease, *n* (%)	3 out of 9 (33.3%)
Solid organ transplantation, *n* (%)	1 out of 9 (11.1%)
Malignancy on chemotherapy, *n* (%)	1 out of 9 (11.1%)
**Antibiotic susceptibility**	
Antibiogram available, *n* (%)	8 out of 9 (88.9%)
Penicillin resistance, *n* (%)	7 out of 7 (100%)
Piperacillin tazobactam resistance, *n* (%)	1 out of 4 (25%)
Tetracycline resistance, *n* (%)	1 out of 4 (25%)
Carbapenem resistance, *n* (%)	1 out of 4 (25%)
Quinolone resistance, *n* (%)	1 out of 6 (16.7%)
Cephalosporine resistant, *n* (%)	1 out of 7 (14.3%)
**Information on infection**	
Concomitant infection, *n* (%)	2 out of 9 (22.2%)
Patients with fever, *n* (%)	7 out of 9 (77.8%)
Patients with sepsis, *n* (%)	6 out of 9 (66.7%)
Patients with organ dysfunction, *n* (%)	2 out of 9 (22.2%)
Patients with shock, *n* (%)	1 out of 9 (11.1%)
Patients requiring ICU care, *n* (%)	2 out of 9 (22.2%)
**Treatment of *E. hermannii* bacteremias**	
Aminoglycosides, *n* (%)	4 out of 8 (50%)
Piperacillin tazobactam, *n* (%)	3 out of 8 (37.5%)
Cephalosporins, *n* (%)	2 out of 8 (25%)
Quinolones, *n* (%)	2 out of 8 (25%)
Trimethoprim sulfomethoxazole, *n* (%)	2 out of 8 (25%)
Carbapenems, *n* (%)	1 out of 8 (12.5%)
Amoxicillin clavulanate, *n* (%)	1 out of 8 (12.5%)
Duration of treatment, median (IQR) in days	14 (14–14)
**Outcome**	
Clinical cure, *n* (%)	6 out of 7 (85.7%)
Deaths due to the infection, *n* (%)	1 out of 7 (14.3%)
Deaths overall, *n* (%)	1 out of 7 (14.3%)

IQR: Intraquartile range, ICU: Intensive care unit.

**Table 2:** Characteristics of patients with *E. hermannii* urinary tract infections. The values show the cases among patients with available data.

Characteristic	Value
Male, *n* (%)	2 out of 3 (66.7%)
Age, median (IQR) in years	54 (43–65)
**Medical history**	
Solid organ transplantation, *n* (%)	1 out of 4 (25%)
**Antibiotic susceptibility**	
Antibiogram available, *n* (%)	4 out of 4 (100%)
Penicillin resistance, *n* (%)	4 out of 4 (100%)
Piperacillin tazobactam resistance, *n* (%)	1 out of 2 (50%)
Cephalosporin resistance, *n* (%)	1 out of 3 (33.3%)
Quinolone resistance, *n* (%)	1 out of 3 (33.3%)
Aminoglycoside resistance, *n* (%)	1 out of 3 (33.3%)
ESBL phenotype, *n* (%)	1 out of 3 (33.3%)
**Information on infection**	
Concomitant infection, *n* (%)	1 out of 4 (25%)
Patients with fever, *n* (%)	2 out of 3 (66.7%)
Patients with sepsis, *n* (%)	2 out of 3 (66.7%)
Patients with organ dysfunction, *n* (%)	1 out of 3 (33.3%)
Patients with shock, *n* (%)	0 out of 3 (0%)
Patients requiring ICU care, *n* (%)	0 out of 3 (0%)
**Treatment of *E. hermannii* bacteremias**	
Cephalosporins, *n* (%)	2 out of 3 (66.7%)
Trimethoprim sulfomethoxazole, *n* (%)	2 out of 3 (66.7%)
Aminoglycosides, n (%)	1 out of 3 (33.3%)
Quinolones, *n* (%)	1 out of 3 (33.3%)
Amoxicillin clavulanate, *n* (%)	1 out of 4 (25%)
Duration of treatment, median (IQR) in days	35 (14–56)
**Outcome**	
Clinical cure, *n* (%)	3 out of 3 (100%)
Deaths overall, *n* (%)	0 out of 3 (0%)

IQR: Intraquartile range, ESBL: Extended spectrum beta-lactamase, ICU: Intensive care unit.
